# Supplementary material for: Shortcuts to adiabaticity for open systems in circuit quantum electrodynamics
Source: Nat Commun. 2022 Jan 10;13:188. doi: 10.1038/s41467-021-27900-6 (PMC8748912; doi:10.1038/s41467-021-27900-6)
Supplement: Supplementary file 1 — Supplementary Information [file 41467_2021_27900_MOESM1_ESM.pdf]

Supplementary Information for Shortcuts to Adiabaticity for Open Systems in Circuit Quantum Electrodynamics

Zelong Yin,[1] Chunzhen Li,[1] Jonathan Allcock,[1] Yicong Zheng,[1]
Xiu Gu,[1] Maochun Dai,[1] Shengyu Zhang,[1] and Shuoming An[1,*]

[1] *Tencent Quantum Laboratory, Tencent, Shenzhen, Guangdong 518057, China*

(Dated: December 13, 2021)

CONTENTS

Supplementary Note 1. Exact Input-output Theory 2

Supplementary Note 2. Single-Mode Counterdiabatic Driving: Short Derivation 5

Supplementary Note 3. Single-Mode Counterdiabatic Driving: Open Quantum Dynamics Approach 5

Supplementary Note 4. Quantum Speed Limit of the CD Driving Protocol 10

Supplementary Note 5. Derivation of the Multi-Mode Optimal Control Protocol 12

Supplementary Note 6. Numerical Optimization and Speed Limit of the MMOC Protocol 15

Supplementary Note 7. Influences of the Large Drive 17

Supplementary Note 8. Propagator Corrections from the Low-pass Filter in the AWG Driving Line 17

Supplementary Note 9. Additional Experimental Data 21

References 31

SUPPLEMENTARY NOTE 1. EXACT INPUT-OUTPUT THEORY

Here we derive the exact input-output formula used to simulate the output signal of the system shown in Fig. 1. Our analysis follows that of Ref. [1], although we additionally account for the distance l from the input capacitor $C_{\rm in}$ to the filter a, which is necessary for the theory to match experimental observations. For wavenumber k, the phase accumulated after passing through this distance is $\theta = k \times l$. We find that this phase has a profound effect on the final output signal. Our goal is to determine how the output field $r_{\rm o}$ responds to

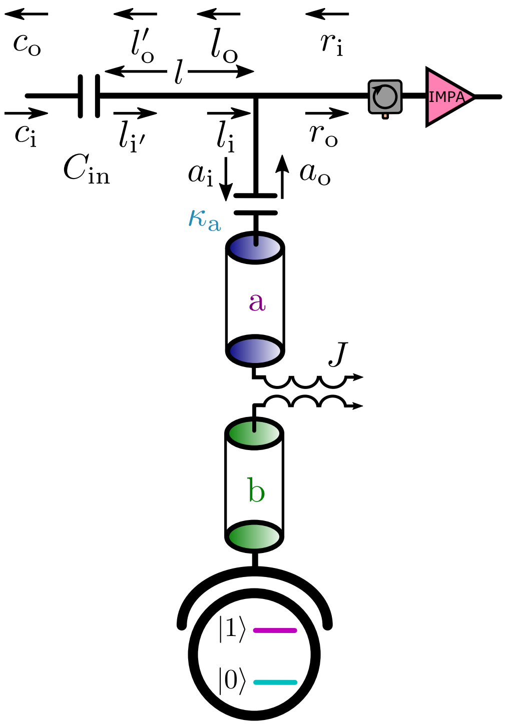

Supplementary Figure 1. **Modes in the system.** The signal network we analyse here includes the feedline, the filter cavity (mode a), the resonator cavity (mode b) and the qubit. In our feed line, there is an input capacitor to reflect the system leakage to the output port. After the filter, there are three circulators and an impedance modified amplifier. All modes and their directions in the calculation are shown.

the input field $c_{\rm i}$ and its interaction with the system, including filter mode a, the resonator mode b and the qubit state. To simplify the calculation, instead of directly including the qubit state, we will account for its effect by modifying other system mode frequencies.

To build the mode network, we start from the most left input port and consider the

transition and reflection of $C_{\rm in}$ as:

$$\begin{aligned} l'_{\rm i} &= (1-\Gamma)c_{\rm i} + \Gamma l'_{\rm o} \\ c_{\rm o} &= \Gamma c_{\rm i} + (1-\Gamma) l'_{\rm o}, \end{aligned} \tag{1}$$

where $\Gamma = \frac{Z_{\rm l}-Z_0}{Z_{\rm l}+Z_0}$ is the reflection coefficient, Z_0 is the impedance of the line and the loaded impedance of $C_{\rm in}$ is $Z_{\rm l} = \frac{1}{i\omega C_{\rm in}}$. As a second step, we consider the effect of the microwave length from $C_{\rm in}$ to the system as:

$$\begin{aligned} l_{\rm i} &= e^{i\theta} l'_{\rm i} \\ l_{\rm o} &= e^{-i\theta} l'_{\rm o}. \end{aligned} \tag{2}$$

After this, the microwave reaches the T connection between the filter and the feedline. Its scattering matrix is:

$$\begin{aligned} l_{\rm o} &= -\frac{1}{3} l_{\rm i} + \frac{2}{3} r_{\rm i} + \frac{2}{3} a_{\rm o} \\ r_{\rm o} &= \frac{2}{3} l_{\rm i} - \frac{1}{3} r_{\rm i} + \frac{2}{3} a_{\rm o} \\ a_{\rm i} &= \frac{2}{3} l_{\rm i} + \frac{2}{3} r_{\rm i} - \frac{1}{3} a_{\rm o}. \end{aligned} \tag{3}$$

Here we assume the impedance of the line connecting the capacitor is also Z_0. Part of the wave in the feedline will drive the filter mode a, which satisfies the input-output formula:

$$a_{\rm o} = a_{\rm i} + \sqrt{\kappa_{\rm a}} a, \tag{4}$$

where $\kappa_{\rm a}$ is the leakage rate of the mode a. Assuming no output reflection ($r_{\rm i} = 0$), the relation between the input field $c_{\rm i}$, the output field $r_{\rm o}$ and the filter mode a is:

$$r_{\rm o} = (1-\Gamma) e^{i\theta} c_{\rm i} + \frac{1 + e^{2i\theta}\Gamma}{2} \sqrt{\kappa_{\rm a}} a. \tag{5}$$

To determine the relation between the input $c_{\rm i}$ and the output $r_{\rm o}$ it suffices to deduce how a depends on $c_{\rm i}$. Under the rotating wave approximation (RWA), the equations of motion in the drive frequency ($\omega_{\rm s}$) rotating frame are:

$$\begin{aligned} \dot{a} &= -i\Delta_{\rm a} a - iJb - \frac{\kappa_{\rm a}}{2} a - \sqrt{\kappa_{\rm a}} a_{\rm i} \\ \dot{b} &= -i\Delta_{\rm b} b - iJa, \end{aligned} \tag{6}$$

where $\Delta_{\rm a(b)} = \omega_{\rm a(b)} - \omega_{\rm s}$ is the detuning of mode $a(b)$ frequency $\omega_{\rm a(b)}$ relative to the drive frequency $\omega_{\rm s}$, and J is the coupling strength between modes a and b. It follows from Eq. 1, 2, 3 and 4 that :

$$a_{\rm i} = \frac{1-\Gamma}{2} e^{i\theta} c_{\rm i} - \frac{(1-e^{i2\theta}\Gamma)}{4} \sqrt{\kappa_{\rm a}} a. \tag{7}$$

Combining Eq. 6 and 7 gives:

$$\begin{aligned} \dot{a} &= -i\Delta_{\mathrm{a}}' a - iJb - \frac{\tilde{\kappa}_{\mathrm{a}}}{2} a - \sqrt{\tilde{\kappa}_{\mathrm{a}}}\tilde{a}_{\mathrm{i}} \\ \dot{b} &= -i\Delta_{\mathrm{b}} b - iJa, \end{aligned} \tag{8}$$

where the effective detuning, leakage rate, and input field of mode a are $\Delta_{\mathrm{a}}' = \Delta_{\mathrm{a}} + \mathrm{Im}(\Gamma e^{i2\theta})\kappa_{\mathrm{a}}/4$, and $\tilde{\kappa}_{\mathrm{a}} = \kappa_{\mathrm{a}}[1 + \mathrm{Re}(\Gamma e^{i2\theta})]/2$, and $\tilde{a}_{\mathrm{i}} = \sqrt{\kappa_{\mathrm{a}}}(1 - \Gamma)e^{i\theta}c_{\mathrm{i}}/(2\sqrt{\tilde{\kappa}_{\mathrm{a}}})$ respectively. Note that Eq. 8 is equivalent, up to redefining various parameters, to Eq. 3 and Eq. 4 of the main text. According to the designed values of l and C_{in}, we estimate $\theta \sim 0.05$ and $\Gamma \sim 0.98 - 0.17i$. To simulate the dynamics of modes a and b, we use a Lindblad master equation with the Hamiltonian:

$$H(t) = \Delta_{\mathrm{b}} b^\dagger b + \Delta_{\mathrm{a}}' a^\dagger a + J(a^\dagger b + b^\dagger a) + i\epsilon^*(t)a - i\epsilon(t)a^\dagger \tag{9}$$

and the Lindblad operator $\sqrt{\tilde{\kappa}_{\mathrm{a}}}a$. The effective driving field $\epsilon(t)$ follows from Eq. 8 and 9 as

$$\epsilon(t) = \sqrt{\tilde{\kappa}_{\mathrm{a}}}\tilde{a}_{\mathrm{i}}, \tag{10}$$

which is averaged in the simulation, given the classical (coherent) input field c_{i}. Substituting this into Eq. 5 gives our final input-output formula:

$$r_{\mathrm{o}} = \frac{2}{\sqrt{\kappa_{\mathrm{a}}}}\epsilon(t) + \frac{1 + e^{i2\theta}\Gamma}{2}\sqrt{\kappa_{\mathrm{a}}}a. \tag{11}$$

To account for the weak nonlinearity of the resonator and the uncertainty in the estimated design parameters, in the simulation we multiply the $\sqrt{\kappa_{\mathrm{a}}}a$ term on the right-hand side of Eq. 11 by a complex coefficient, chosen to fit the simulation to experimental data.

Physical interpretation. Because the filter mode a driven by $\epsilon(t)$ can be solved using the Lindblad master equation, we can determine the output mode r_{o} once we know the driving waveform $\epsilon(t)$. The physical meaning of Eq. 11 can be interpreted as follows. The factor 2 before $\epsilon(t)$ means only half of the input mode c' is used to drive mode a. The signal that finally reaches the output port is twice the driving. The $e^{i2\theta}\Gamma$ term means half of the leakage of a directly goes to the output port, and the other half will go to the input side and be reflected by C_{in}. Finally, these two branches interfere with each other and contribute a complex factor between the input and the system leakage.

SUPPLEMENTARY NOTE 2. SINGLE-MODE COUNTERDIABATIC DRIVING: SHORT DERIVATION

Here we give a simple, short derivation of the counterdiabatic (CD) driving (Eq. 1 in the main text) for a single driven bosonic mode coupled to a cold bath, based on a mean-field approximation. We leave a rigorous derivation to Supplementary Note (SN) 3.

As we will see in SN 3, the bosonic mode under consideration can be well approximated by a coherent state, and thus we can use a mean-field approximation for the Heisenberg picture bosonic field $a(t)$, *i.e.* $\alpha(t) = \langle a(t) \rangle$. Following SN 1, the dynamics are given by the Langevin equation in the drive frame of frequency ω_{s}:

$$\dot{\alpha} = -i\Delta_{\mathrm{r}}\alpha - \frac{\kappa}{2}\alpha - \epsilon(t) \tag{12}$$

where $\Delta_{\mathrm{r}} \equiv \omega_{\mathrm{r}} - \omega_{\mathrm{s}}$ is the cavity-drive detuning, κ is the damping rate due to coupling to the readout line, and $\epsilon(t)$ is the effective drive field. The instantaneous equilibrium state is obtained by setting $\dot{\alpha} = 0$. We denote this instantaneous equilibrium state as:

$$\bar{\alpha}(t) = \frac{i\epsilon(t)}{\Delta_{\mathrm{r}} - i\kappa/2}, \tag{13}$$

If the drive field is varied slowly enough, the adiabatic theorem guarantees that $\bar{\alpha}(t)$ be the solution of Eq. 12. Define the instantaneous diabatic excitation $\delta(t) = \alpha(t) - \bar{\alpha}(t)$. It follows from Eq. 12, and Eq. 13 and its time derivative, that the dynamics of $\delta(t)$ satisfies:

$$\dot{\delta}(t) = -i(\Delta_{\mathrm{r}} - i\kappa/2)\delta(t) - \epsilon_{\mathrm{CD}}(t) + \left(\epsilon(t) - i\frac{\dot{\epsilon}(t)}{\Delta_{\mathrm{r}} - i\kappa/2}\right). \tag{14}$$

where ϵ_{CD} is the (new) CD driving. From the boundary conditions $\delta(0) = 0$ and $\dot{\delta}(0) = 0$, we obtain the desired CD driving as:

$$\epsilon_{\mathrm{CD}}(t) = \epsilon(t) - i\frac{\dot{\epsilon}(t)}{\Delta_{\mathrm{r}} - i\kappa/2}. \tag{15}$$

Then, for an arbitrarily drive $\epsilon(t)$, the instantaneous equilibrium state $\bar{\alpha}(t)$ is always the exact dynamic solution of Eq. 12.

SUPPLEMENTARY NOTE 3. SINGLE-MODE COUNTERDIABATIC DRIVING: OPEN QUANTUM DYNAMICS APPROACH

In this section, we give rigorous derivations of CD driving for a single driven-dissipative bosonic mode, based on two approaches: (i) Lindblad dynamics [2] and (ii) an adiabatic

shortcut of the decoherence free subspace (DFS) [3]. These results justify the mean-field approximation assumed in SN 2, and give additional insight into the adiabatic dynamics of our system.

In what follows, we set $\hbar = 1$. After rotating wave approximation, the Hamiltonian in the driving frame is:

$$H(t) = \Delta_{\rm r} a^\dagger a - i\left(\epsilon(t) a^\dagger - \epsilon^*(t) a\right). \tag{16}$$

where the cavity-drive detuning $\Delta_{\rm r} = \omega_{\rm r} - \omega_{\rm s}$ depends on the qubit state in the dispersive regime, and $\epsilon(t)$ is the effective drive amplitude. The transmission line is viewed as a channel for both driving and dissipation, so the dynamics for the cavity density matrix ρ is described by the master equation:

$$\begin{aligned}\dot\rho(t) &= \mathcal{L}(t)\rho(t)\\ &= -i[H(t),\rho(t)] + \kappa\mathcal{D}[a]\rho(t)\end{aligned} \tag{17}$$

where the dissipator is $\mathcal{D}[a]\rho(t) = a\rho(t)a^\dagger - \frac{1}{2}\{\rho(t), a^\dagger a\}$. Here, only photon decay is considered, since at the effective temperature $T_{\rm mxc} = 75\,\mathrm{mK}$, the average photon population is $N \approx 0.015 \ll 1$ at readout frequency $\omega_{\rm s} \approx 2\pi \times 6.5\,\mathrm{GHz}$.

Lindblad dynamics approach. In the adiabatic approximation for open systems [4], in the limit where the Liouvillian $\mathcal{L}(t)$ is slowly varying, the density matrix ρ evolves independently in each generalized eigenspace of $\mathcal{L}(t)$. In other words, ρ can be decomposed into a direct sum of components, one for each independently evolving Jordan block of $\mathcal{L}(t)$. The adiabaticity can be made exact by adding a CD Hamiltonian $H_{\rm CD}$ which suppress the inertial part of $\mathcal{L}(t)$ that causes transitions between different Jordan blocks [2].

To determine $H_{\rm CD}$, we first we find a superoperator $\hat{\mathcal{O}}(t)$ that transforms $\mathcal{L}(t)$ into Jordan canonical form (JCF). That is, with respect to a certain (not necessarily Hermitian) basis for the density matrix $B = \{\rho_1, \rho_2, \ldots\}$, we have

$$\hat{\mathcal{O}}(t)^{-1}\mathcal{L}(t)\hat{\mathcal{O}}(t) = \mathrm{diag}(J_1(t), J_2(t), \ldots) \tag{18}$$

where $J_i(t)$ are the Jordan blocks of size $n_i \times n_i$. Second, we transfer to the adiabatic frame defined by $\rho'(t) = \hat{\mathcal{O}}(t)^{-1}\rho(t)$ and show that the non-JCF part of the new Lindblad superoperator $\mathcal{L}'(t)$, *i.e.* $\dot\rho'(t) = \mathcal{L}'(t)\rho'(t)$, can be exactly cancelled by adding a specific CD driving Hamiltonian $H_{\rm CD}(t)$ to the system.

In the first step, we choose $\hat{\mathcal{O}}(t)$ to take the form of a displacement superoperator $\hat{\mathcal{D}}(\alpha(t))\rho(t) = D(\alpha(t))\rho(t)D(\alpha(t))^{-1}$ where $D(\alpha) \equiv \exp(\alpha a^\dagger - h.c.)$ is the displacement operator [5]. Using the fact that $\hat{\mathcal{D}}(\alpha(t))a = a - \alpha(t)$, it is straightforward to show that

$$\mathcal{L}_{\mathcal{J}}(t)\rho(t) \equiv \hat{\mathcal{D}}(\alpha(t))^{-1}\mathcal{L}(t)\hat{\mathcal{D}}(\alpha(t))\rho(t) \tag{19}$$

$$= -i[H_{\mathrm{J}}(t), \rho(t)] + \kappa\mathcal{D}[a]\rho(t) \tag{20}$$

$$H_{\mathrm{J}}(t) = \Delta_{\mathrm{r}} a^\dagger a + [\Delta_{\mathrm{r}}\alpha(t) - \frac{i\kappa}{2}\alpha(t) - i\epsilon(t)]a^\dagger + h.c. \tag{21}$$

Choosing $\alpha(t) = \bar{\alpha}(t) \equiv i\epsilon(t)/(\Delta_{\mathrm{r}} - i\kappa/2)$ – *i.e.* precisely the instantaneous equilibrium state of SN 2, Eq. 13 – eliminates the time-dependent driving term in $H_{\mathrm{J}}(t)$. Thus, in the adiabatic frame defined by $D(\bar{\alpha}(t))$, the Liouvillian $\mathcal{L}_{\mathcal{J}}(t) = \mathcal{L}_{\mathcal{J}}$ is time-independent, so a fixed basis B can be chosen in which $\mathcal{L}_{\mathcal{J}}$ is in JCF.

In the second step, in the adiabatic frame $\rho'(t) = \hat{\mathcal{D}}(\bar{\alpha}(t))^{-1}\rho(t)$ we have

$$\begin{aligned} \dot{\rho}'(t) &= -i[i\dot{D}(\bar{\alpha})^{-1}D(\bar{\alpha}), \rho'(t)] + \hat{\mathcal{D}}(\bar{\alpha})^{-1}\mathcal{L}(t)\hat{\mathcal{D}}(\bar{\alpha})\rho'(t) \\ &= -i[i\dot{D}(\bar{\alpha})^{-1}D(\bar{\alpha}), \rho'(t)] + \mathcal{L}_{\mathcal{J}}\rho'(t). \end{aligned} \tag{22}$$

i.e. the dynamics are exactly in JCF except for an inertial Hamiltonian $H_{\mathrm{i}} = i\dot{D}(\bar{\alpha})^{-1}D(\bar{\alpha})$ which mixes the Jordan blocks of $\mathcal{L}_{\mathcal{J}}$. Adding an additional CD term $\mathcal{L}_{\mathrm{CD}}\ \rho = -i[H_{\mathrm{CD}}, \rho]$ to $\mathcal{L}$ exactly cancels H_{i} if $\hat{\mathcal{D}}(\bar{\alpha})^{-1}H_{\mathrm{CD}} = -H_{\mathrm{i}}$. That is, if

$$H_{\mathrm{CD}}(t) = i\dot{D}(\bar{\alpha}(t))D(\bar{\alpha}(t))^{-1} \tag{23}$$

$$= -\frac{\dot{\epsilon}(t)}{\Delta_{\mathrm{r}} - i\kappa/2}a^\dagger + h.c. \tag{24}$$

consistent with SN 2, Eq. 15.

Decoherence free subspace approach. The time-dependent decoherence free subspace (DFS) is a subspace of the full system Hilbert space, in which the open system dynamics is unitary and quasi-steady, *i.e.* its instaneous motion is generated by an effective Hamiltonian H_{eff} defined within the DFS. By identifying the time-dependent DFS of our system, we derive the CD driving (Eq. 24) and compare it to the Lindblad dynamics approach.

Following the definition in [3], for system dynamics described by a Lindblad master equation $\dot{\rho} = \mathcal{L}(t)\rho = -i[H(t), \rho] + \sum_k \mathcal{D}[a_k(t)]\rho$ where the Lindblad operators $a_k(t)$ have possible time dependence, the time-dependent DFS is the space spanned by a set of orthonormal states $\{|\phi_j(t)\rangle\}$, satisfying: (i) the basis states $|\phi_j(t)\rangle$ are degenerate eigenstates

of any Lindblad operator, *i.e.* $a_k(t)|\phi_j(t)\rangle = c_k(t)|\phi_j(t)\rangle\ \forall j,k$; (ii) the DFS is closed under the effective Hamiltonian $H_{\rm eff}(t) \equiv H(t) + i/2\sum_k c_k^*(t)a_k(t) + h.c.$, *i.e.* $H_{\rm eff}$ acting on a state in the DFS results in a state in the DFS. For a single lossy mode described by Eq. 17, the DFS exists and is spanned by the single state $|\bar\alpha(t)\rangle$ for $\bar\alpha(t) = i\epsilon(t)/(\Delta_{\rm r} - i\kappa/2)$, and $H_{\rm eff}$ takes the form of a displaced oscillator $H_{\rm eff}(t) = \Delta_{\rm r}(a^\dagger - \bar\alpha^*(t))(a - \bar\alpha(t))$.

Suppose evolution of the DFS is given by the unitary transformation $U(t)$, *i.e.* $U(t)|\phi_j(0)\rangle = |\phi_j(t)\rangle$. By direct analogy with closed system CD driving, we can transform to the adiabatic frame defined by $U(t)$ and cancel diabatic excitations out of the DFS by adding a CD Hamiltonian $H_{\rm CD}(t) = i\dot U(t)U(t)^\dagger$. In our example, the natural choice for $U(t)$ is the displacement $D(\bar\alpha(t))$, from which we can derive the CD Hamiltonian $H_{\rm CD} = i\dot D(\bar\alpha(t))D(\bar\alpha(t))^{-1}$, equivalent to Eq. 24 obtained from the Lindblad dynamics approach.

Comments.

Steady states and adiabatic timescale. In the adiabatic frame, the Liouvillian $\mathcal{L}_{\mathcal{J}}$ in Eq. 21 takes the form $\mathcal{L}_{\mathcal{J}}\rho = -i[\Delta_{\rm r}a^\dagger a, \rho] + \kappa\mathcal{D}[a]\rho$. Its eigenvalues can be found by observing

$$\mathcal{L}_{\mathcal{J}}|m\rangle\langle n| = [-i\Delta_{\rm r}(m-n) - \frac{\kappa}{2}(m+n)]|m\rangle\langle n| + \kappa\sqrt{mn}|m-1\rangle\langle n-1| \tag{25}$$

where $|n\rangle$ are the Fock states, indicating $\mathcal{L}_{\mathcal{J}}$ is upper triangular in the subspace spanned by $\{|0\rangle\langle j|, |1\rangle\langle j+1|, |2\rangle\langle j+2|, \ldots\}$ (or their Hermitian conjugates) for natural numbers j. Hence, $\mathcal{L}_{\mathcal{J}}$ has non-degenerate eigenvalues $e_{j,k} = i\Delta_{\rm r}j - \kappa(j/2+k),\ k = 0,1,2,\ldots$ (or their complex conjugates) in each subspace and can be exactly diagonalized. We note that the only steady state of $\mathcal{L}_{\mathcal{J}}$, *i.e.* the eigenstate of $\mathcal{L}_{\mathcal{J}}$ with zero eigenvalue, is given by $j = k = 0$, which is the vacuum state $|0\rangle$ in the adiabatic frame or the coherent state $|\alpha(t)\rangle$ in the lab frame.

We also comment on the timescale required for the adiabatic approximation to hold in open systems, following the results of [4]. Analogously to closed quantum systems, a sufficient condition for adiabatic evolution of an open system is

$$\max_{0<t<t_{\rm f}} |\langle\tilde\rho_{j',k'}(t), \dot\rho_{j,k}(t)\rangle| \ll |e_{j,k} - e_{j',k'}|,\ \forall j,k,j',k' \tag{26}$$

where $t_{\rm f}$ is the total evolution time, $\langle u,v\rangle \equiv {\rm tr}(u^\dagger v)$ defines the inner product, $\rho_{j,k}(t)$ are (lab-frame) eigenstates of $\mathcal{L}(t)$ with eigenvalues $e_{j,k}$, and $\tilde\rho_{j,k}(t)$ are eigenstates of $\mathcal{L}(t)^\dagger$. Here the adjoint $\mathcal{L}(t)^\dagger$ is defined as the superoperator that satisfy $\langle\mathcal{L}(t)^\dagger u, v\rangle = \langle u, \mathcal{L}(t)v\rangle,\ \forall u,v$. The LHS is hard to evaluate in practice, and a crude estimate is obtained by setting

$\langle \tilde{\rho}_{j',k'}(s), d\rho_{j,k}(s)/ds \rangle \sim 1$ for normalized time $s \equiv t/t_{\mathrm{f}}$. The adiabatic condition for the total time t_{f} is then derived as

$$t_{\mathrm{f}} \gg \frac{1}{\min_{j,k,j',k'}(|e_{j,k} - e_{j',k'}|)} = \frac{1}{\min(\sqrt{\Delta_{\mathrm{r}}^2 + \kappa^2/4}, \kappa)}. \tag{27}$$

For Δ_{r} comparable to κ, which is a usual experimental scenario, the adiabatic condition is $t_{\mathrm{f}} \gg \kappa^{-1}$, making STA useful for fast protocols operating within unit lifetimes. This adiabatic condition is verified in Fig. 10, where $\sin^2$-shaped pulses are applied for different durations t_{f} and $\sin^2$-shaped output signals are observed only for $t_{\mathrm{f}} > 10\kappa^{-1} \approx 600\,\mathrm{ns}$.

DFS from Lindblad dynamics. The derivation of CD driving from both Lindblad dynamics and the DFS approach relies on switching to the adiabatic frame defined by $D(\bar{\alpha}(t))$, *i.e.* $|\phi'\rangle = D(\bar{\alpha}(t))^{\dagger}|\phi\rangle$. We note that the DFS of our system (*i.e.* the coherent state $|\bar{\alpha}(t)\rangle$) is the vacuum state $|0\rangle$ in the adiabatic frame - the only steady eigenstate (*i.e.* having an eigenvalue with a non-negative real part) of $\mathcal{L}_{\mathcal{J}}$. As a result, the steady eigenspace of the Liouvillian $\mathcal{L}(t)$ is equivalent to the DFS, whereas this is not true in general since purity of these steady states requires a zero-temperature approximation or negligible thermal photon number $N(\omega) \ll 1$ in the frequency band of interest. For bosonic modes in the high temperature regime ($N(\omega) \sim 1$ or $N(\omega) \gg 1$), CD driving is still possible by the Lindblad dynamics approach even though the pure-state DFS does not exist. In this case, although CD driving does not prevent heating into the steady thermal state in the adiabatic frame, it ensures fast transport of this steady state, which is still of practical interest.

Mean Field Approximation. Here we show that the single driven-dissipative mode remains in a coherent state, which justifies the mean-field approximation used in SN 2. For open quantum systems, the coherent state is known to be the consequence of the zero-temperature approximation of the environment [6]. Specifically, in the frame defined by a general displacement $D(\alpha(t))$, the dynamics in Eq. 22 can be rewritten as

$$\dot{\rho}' = -i[\Delta_{\mathrm{r}} a^{\dagger} a + (-i\dot{\alpha} + \Delta_{\mathrm{r}}\alpha - i\frac{\kappa}{2}\alpha - i\epsilon)a^{\dagger} + h.c.\ , \rho'] + \kappa\mathcal{D}[a]\rho'. \tag{28}$$

Choosing $\alpha(t)$ that satisfies the Langevin dynamics (Eq. 12) thus eliminates the driving term. Consequently, the system stays in the vacuum state in the displaced frame, corresponding to the coherent state $|\alpha(t)\rangle$ in the lab frame.

SUPPLEMENTARY NOTE 4. QUANTUM SPEED LIMIT OF THE CD DRIVING PROTOCOL

In this section, we discuss the Quantum Speed Limit (QSL) for a driven-dissipative bosonic mode, and show that our CD driving protocol reaches optimal quantum efficiency among all possible experimental controls.

For open quantum systems, the QSL can be formulated as a geometric constraint, *i.e.* the total length of the system's trajectory is bounded below by the geodesic connecting its initial and final states, where the geometry is defined in terms of the Bures metric [7] for density matrices. This metric is interpreted as the statistical distinguishability between neighbouring quantum states, expressed in terms of the quantum generalization of Fisher information, *i.e.* the Fisher information maximized over all choices of quantum measurements [8]. For our system, the dynamics can be equivalently described by a unitary operator, generated by an effective Hamiltonian $H_{\text{eff}}(t) = i\dot{U}(t)U(t)^\dagger$. This reduces the QSL to the Mandelstam-Tamm (MT) bound [9]:

$$\arccos|\langle\phi_{\text{i}}|\phi_{\text{f}}\rangle| \leq \int_{t_{\text{i}}}^{t_{\text{f}}} \sqrt{\langle\Delta H_{\text{eff}}^2(t)\rangle} dt \tag{29}$$

where $|\phi_{i(f)}\rangle$ is the initial(final) state and $\Delta H_{\text{eff}} = H_{\text{eff}} - \langle H_{\text{eff}}\rangle$. Geometrically, the LHS of Eq. 29 is the Bures length s_{Bures} of the geodesic joining the initial and final state and the RHS is the integrated total length of the system trajectory whose velocity is given by $ds_{\text{Bures}}/dt = \sqrt{F_{\text{Q}}(t)/4} = \sqrt{\langle\Delta H_{\text{eff}}^2(t)\rangle}$ [10]. Here, $F_{\text{Q}}(t)$ is the quantum Fisher information. We define the quantum efficiency of our protocol to be:

$$\eta \equiv \frac{\arccos|\langle\phi_{\text{i}}|\phi_{\text{f}}\rangle|}{\int_{t_{\text{i}}}^{t_{\text{f}}} \sqrt{\langle\Delta H_{\text{eff}}^2(t)\rangle} dt} \tag{30}$$

As shown in Eq. 28, for a general driving $\epsilon(t)$ and the system initialized in the ground state, the dynamics is described by the displacement operator, *i.e.* $U(t) = D(\alpha(t))$ where $\alpha(t)$ is the solution to the Langevin equation $\dot{\alpha}(t) = (-i\Delta_{\text{r}} - \kappa/2)\alpha(t) - \epsilon(t)$ (SN 3, Eq. 28). We note that $U(t)$ can generate arbitrary dynamics in the space orthogonal to the system state $|\phi(t)\rangle = |\alpha(t)\rangle$, but the extra freedom can be shown to have no contribution to the uncertainty $\langle\Delta H_{\text{eff}}^2(t)\rangle$. With this choice of $U(t)$ it is straightforward to show that $H_{\text{eff}}(t) = i\dot{\alpha}(t)a^\dagger + h.c.$ and $\sqrt{\langle\Delta H_{\text{eff}}^2(t)\rangle} = |\dot{\alpha}(t)|$. For CD driving, $\sqrt{\langle\Delta H_{\text{eff}}^2(t)\rangle}$ is simply the added drive $|\epsilon_{\text{CD}}(t) - \epsilon(t)|$, which provides the resource for adiabatic speedup in view of the energy-time uncertainty principle. Identifying $|\phi_{i,f}\rangle = |\alpha_{i,f}\rangle$ and applying the triangle

inequality, we obtain

$$\eta \leq \frac{\arccos(e^{-|\alpha_f-\alpha_i|^2/2})}{|\alpha_f-\alpha_i|}. \tag{31}$$

with equality achieved by straight-line trajectories – made possible by CD driving – in

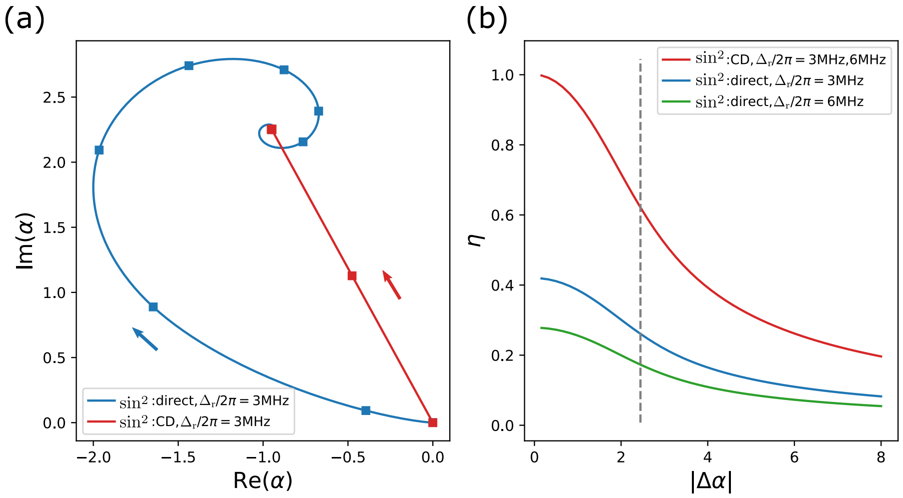

Supplementary Figure 2. **Quantum Efficiency of the CD Driving.** (a) Mean field trajectory $\alpha(t)$ for a constant drive preceded by a $t_f = 100\,\text{ns}$ $\sin^2$ ringup, with direct or CD driving. Markers are plotted every 50 ns. CD driving maximizes quantum efficiency by finding the shortest path towards the final state. (b) Quantum efficiency η for resonator-drive detunings $\Delta_r/2\pi = 3\,\text{MHz},\ 6\,\text{MHz}$, plotted for direct (blue for $\Delta_r/2\pi = 3\,\text{MHz}$, green for $\Delta_r/2\pi = 6\,\text{MHz}$) and CD driving (red for both detunings). Direct driving with higher detuning is less efficient as it induces spiral trajectories with greater length. The gray line shows the efficiencies for the example in (a).

phase space (see Fig. 2 a). The spiral trajectory $\alpha(t)$ in Fig. 2 a is calculated from Eq. 12 with parameters $\Delta_r/2\pi = 3\,\text{MHz}$, $\kappa^{-1} = 62.88\,\text{ns}$. Fig. 2 b shows η as a function of target displacement $|\Delta\alpha| = |\alpha_f - \alpha_i|$ for direct and CD driving with two resonator-drive detunings Δ_r. For both detunings, the CD driving reaches the optimal quantum efficiency experimentally (as given by the right hand side of Eq. 31). In particular, it saturates the MT bound (*i.e.* $\eta \to 1$) in the small driving limit $|\Delta\alpha| \to 0$. The inefficiency at large $|\Delta\alpha|$ can be explained by the inability to create direct driving to higher-level Fock states, which is a general issue in applying the MT bound for systems with large numbers of energy levels like the bosonic system we consider. Nevertheless, CD driving achieves optimal quantum

efficiency within the space of all available pulses, making it favourable for experimental realisation.

SUPPLEMENTARY NOTE 5. DERIVATION OF THE MULTI-MODE OPTIMAL CONTROL PROTOCOL

In this section, we derive the multi-mode optimal control (MMOC) protocol used in the main text, which takes the hybrid frequencies of multiple oscillators as input, and generates a single-port waveform that puts these lossy bosonic modes into thermal equilibrium at a desired final time t_f. We first present a general framework which can be applied to multiple port driving, and then analyse the simpler single port case which is analytically and experimentally more tractable, and sufficient for our needs in the main text.

General multiple-port framework. Consider n linear bosonic modes $\{a_i\}_{i=1}^n$ with frequencies ω_i and linear couplings J_{ij} between modes a_i and a_J. Each mode is coupled to a feedline with strength κ_i and driven by an input field c_i at frequency ω_d. In general, the fields c_i can be linearly dependent if they come from the same feedline. In the rotating frame with frequency ω_d and after rotating wave approximation (RWA), the system Hamiltonian has the form

$$H_\mathrm{S} = \sum_{i=1}^{n} \Delta_i a_i^\dagger a_i + \sum_{i=1}^{n} \sum_{j=1, j\neq i}^{n} (J_{ij} a_i^\dagger a_j + h.c.) \tag{32}$$

where $\Delta_i = \omega_i - \omega_\mathrm{s}$ is the ith detuning. In the Heisenberg picture, following the input-output formalism [11], the Langevin dynamics for the ith mode is given by $\dot{a}_i = -i[a_i, H_\mathrm{S}] - (\kappa_i/2)a_i - \sqrt{\kappa_i} c_i$. Adopting the mean-field approximation $\alpha_i \equiv \langle a_i \rangle$ for all bosonic modes and defining the effective drive $\epsilon_i \equiv \sqrt{\kappa_i}\langle c_i \rangle$, we can rewrite the Langevin dynamics in matrix form:

$$\frac{d}{dt}\alpha + \mathbf{i\Omega}\cdot\alpha = -\epsilon,\ \mathbf{\Omega} = \begin{pmatrix} \Delta_1 - i\kappa_1/2 & J_{12} & \dots & J_{1n} \\ J_{12}^* & \Delta_2 - i\kappa_2/2 & \dots & J_{2n} \\ \dots & \dots & & \dots \\ J_{1n}^* & J_{2n}^* & \dots & \Delta_n - i\kappa_n/2 \end{pmatrix} \tag{33}$$

where Ω is the (complex) frequency matrix, $\alpha = (\alpha_\mathbf{1}, \dots, \alpha_\mathbf{n})^\mathbf{T}$ is the column vector of the mean fields, and $\epsilon(\mathbf{t}) = (\epsilon_\mathbf{1}(\mathbf{t}), \dots, \epsilon_\mathbf{n}(\mathbf{t}))^\mathbf{T}$ is the column vector of the effective drives. Ω can be diagonalized as $\Omega = O^{-1}\Omega_\mathrm{D} O$, where $\Omega_\mathrm{s} = \mathrm{diag}(\Delta_1' - i\kappa_1'/2, \dots, \Delta_n' - i\kappa_n'/2)$ defines the hybrid detunings and linewidths as Δ_i', κ_i'. We use step function driving in our protocol: the

time between initial time t_0 and final time $t_\mathrm{f} = t_m$ is divided into m equal-length intervals, over each of which the drive strength is constant. *i.e.* for each drive ϵ_i:

$$\begin{aligned} \epsilon_i(t < t_0) &= \epsilon_{i0} \\ \epsilon_i(t_{j-1} < t < t_j) &= \epsilon_{ij}, \quad j \in \{1, 2, 3, \dots, m\} \\ \epsilon_i(t > t_\mathrm{f}) &= \epsilon_{i\mathrm{f}}. \end{aligned} \tag{34}$$

where the $\epsilon_{i0}, \epsilon_{ij}, \epsilon_{if}$ are constants. Our goal is to put $\alpha(\mathbf{t})$ into the target equilibrium state $\alpha_\mathrm{f} = \mathbf{i}\boldsymbol{\Omega}^{-1}\epsilon_\mathrm{f}$ at final time t_f, starting from initial equilibrium state $\alpha_\mathbf{0} = \mathbf{i}\boldsymbol{\Omega}^{-1}\epsilon_\mathbf{0}$. The propagator and general solution of differential equation Eq. 33 are

$$D(t - t') = e^{-i\Omega(t-t')}\theta(t - t') \tag{35}$$

$$\alpha(\mathbf{t}) = e^{-i\Omega(t-t_0)}\alpha(\mathbf{t_0}) - \int_{\mathbf{t_0}}^{\mathbf{t}} \mathbf{e}^{-\mathbf{i}\boldsymbol{\Omega}(\mathbf{t}-\mathbf{t}')}\epsilon(\mathbf{t}')\mathbf{dt}' \ (\mathbf{t} > \mathbf{t_0}), \tag{36}$$

where θ is the step function. Using $\Omega = O^{-1}\Omega_D O$ and Eq. 36, our goal can be achieved by solving the equations

$$\sum_{i=1}^{n}\sum_{j=1}^{m} O_{ki}G_{kj}\epsilon_{ij} = \frac{1}{i\tilde{\Delta}'_k}\sum_{i=1}^{n} O_{ki}\left(\epsilon_{i\mathrm{f}} - \epsilon_{i0}e^{-i\tilde{\Delta}'_k(t_\mathrm{f}-t_0)}\right) \tag{37}$$

$$G_{kj} \equiv \int_{t_{j-1}}^{t_j} e^{-i\tilde{\Delta}'_k(t_\mathrm{f}-t')}dt', \tag{38}$$

for ϵ_{ij}, where $\tilde{\Delta}'_k = \Delta'_k - i\kappa'_k/2$ is the complex hybrid detuning. Treating the piece-wise driving ϵ_{ij} as a vector ϵ_l of dimension $n\times m$ and defining the $n\times mn$ matrix $M_{kl} \equiv O_{ki}G_{kj}$ ($l = 1, 2, \dots, mn$), Eq. 37 reduces to the linear equations

$$\sum_{l=1}^{mn} M_{kl}\epsilon_l = y_k, \;\; y_k \equiv \frac{1}{i\tilde{\Delta}'_k}\sum_{i=1}^{n} O_{ki}\left(\epsilon_{i\mathrm{f}} - \epsilon_{i0}e^{-i\tilde{\Delta}'_k(t_\mathrm{f}-t_0)}\right) \tag{39}$$

If complete information of O or Ω are given, the general solution of Eq. 39 can be found by performing a singular value decomposition (SVD) of the matrix M. We concentrate instead on the case of single-port driving, which is considerably simpler.

Single port driving. In the special case of single-port driving, all drivings $\epsilon_i(t)$ are linearly dependent, and Eq. 34 reduces to

$$\begin{aligned} \epsilon_i(t < t_0) &= c_\mathrm{i}\epsilon_0 \\ \epsilon_i(t_{j-1} < t < t_j) &= c_\mathrm{i}\epsilon_j, \quad j \in \{1, 2, 3, \dots, m\} \\ \epsilon_i(t > t_\mathrm{f}) &= c_\mathrm{i}\epsilon_\mathrm{f}. \end{aligned} \tag{40}$$

for constant coefficients c_{i}, a single-port driving vector ϵ_j, and boundary conditions ϵ_0, ϵ_{f}. In this case the $\sum_i O_{ki} c_i$ terms in Eq. 37 cancel, to give

$$\mathbf{G}\cdot\epsilon = \mathbf{y}, \quad \mathbf{y_k} \equiv \frac{\mathbf{1}}{\mathbf{i}\tilde{\boldsymbol{\Delta}}'_{\mathbf{k}}}(\epsilon_{\mathrm{f}} - \epsilon_{\mathbf{0}}\mathbf{e}^{-\mathbf{i}\tilde{\boldsymbol{\Delta}}'_{\mathbf{k}}(\mathbf{t}_{\mathrm{f}}-\mathbf{t_0})}), \tag{41}$$

which takes the form of a linear constraint on ϵ.

Eq. 41 can be similarly solved via SVD of the $n\times m$ matrix G, *i.e.* $G = U\cdot D\cdot V$ for unitary matrices U, V and diagonal matrix $D = (\mathrm{diag}(s_1,\ldots,s_n), 0_{n\times(m-n)})$ ($m \geq n$, 0 is the zero matrix). This gives the general form of ϵ as

$$\epsilon = \sum_{\mathbf{i=1}}^{\mathbf{n}} \frac{(\mathbf{U^{-1}y})_{\mathbf{i}}}{\mathbf{s_i}}\mathbf{V_i^{-1}} + \sum_{\mathbf{i=n+1}}^{\mathbf{m}} \mathbf{x_i}\mathbf{V_i^{-1}}, \tag{42}$$

where $x_{n+1},\ldots,x_m$ are free complex parameters and V_i^{-1} is the ith column of V^{-1}, which can be chosen to optimize a user-defined objective function such as the maximum power output of the pulse (see SN 6). We note that in the single-port driving case, the only input to the protocol is the complex detuning $\tilde{\Delta}'_i$, which is simpler to measure experimentally than the multi-port driving case where full information of $\mathbf{\Omega}$ is required.

Experimental implementation. Our single-port driving experiment in the main text corresponds to

$$\mathbf{a} = (\mathbf{a_0}, \mathbf{b_0}, \mathbf{a_1}, \mathbf{b_1})^{\mathbf{T}}, \epsilon = (\epsilon, \mathbf{0}, \epsilon, \mathbf{0})^{\mathbf{T}}, \mathbf{\Omega} = \begin{pmatrix} \Delta_{\mathrm{a}} - i\kappa_{\mathrm{a}}/2 & J & 0 & 0 \\ J & \Delta_{b,0} & 0 & 0 \\ 0 & 0 & \Delta_{\mathrm{a}} - i\kappa_{\mathrm{a}}/2 & J \\ 0 & 0 & J & \Delta_{b,1} \end{pmatrix} \tag{43}$$

where a_i, b_i are the Purcell filter and readout resonator field conditioned on qubit state $i = 0, 1$, $\Delta_{b,i}$ is the resonator detuning conditioned on qubit state, and ϵ is the effective driving on the filter port. The drive constants are $c_1 = c_3 = 1, c_2 = c_4 = 0$ in Eq. 40, and the solution to Eq. 42 determines the two quadratures of the driving function which, after optimization over the parameters x_i (discussed in SN 6), yields the waveform used in Fig. 3 of the main text.

Applications. Two applications of the class of waveforms derived above are fast equilibration of the readout cavity and Purcell filter and the fast reset of them to the vacuum state. In the first case, we set $\epsilon_0 = 0$ and ϵ_{f} in Eq. 41 to be the constant drive amplitude after t_{f}. In the second case, reverse ϵ_0 and ϵ_{f}. Unlike the continuous driving pulse in the

CD case, the MMOC protocol results in many pulse jumps. In SN 8, we estimate the effect of the distortion induced by the filter in the AWG and confirm we can still use the MMOC pulses safely.

SUPPLEMENTARY NOTE 6. NUMERICAL OPTIMIZATION AND SPEED LIMIT OF THE MMOC PROTOCOL

This section covers various numerical aspects of the single-port MMOC protocol of SN 5, including optimization over the maximum power needed, the speed limit of the protocol given limited output power, and the computational complexity of calculating the desired pulse.

Energy consumption. The total energy consumption (up to an overall constant) of our pulse in Eq. 42 is, due to unitarity of V^{-1},

$$E(\{x_i\}) \equiv \langle \epsilon(\{\mathbf{x_i}\}), \epsilon\ (\{\mathbf{x_i}\})\rangle = \sum_{\mathbf{i=1}}^{\mathbf{n}} \left| \frac{(\mathbf{U^{-1}y})_\mathbf{i}}{\mathbf{s_i}} \right|^\mathbf{2} + \sum_{\mathbf{i=n+1}}^{\mathbf{m}} |\mathbf{x_i}|^\mathbf{2}, \tag{44}$$

where $\langle \mathbf{u}, \mathbf{v}\rangle$ denotes the inner product. From Eq. 44 we see that the minimum energy solution E_{min} is obtained by setting $x_i = 0$.

Minimizing the maximum power output. Given the output power limitations of the microwave devices, it is desirable to minimize the maximum output power $P_{\text{max}}(\{x_i\}) \equiv \max_i(|\epsilon_i(\{x_i\})|^2)$ of the pulse. To achieve this, we numerically minimize P_{max} (as a function of free parameters x_i from Eq. 42) using a differential evolution algorithm. The resulting optimized MMOC pulses for both the ring-up and reset stage are those used in the main text.

Fig. 3 shows the numerical results of P_{max} (in dB) in units of the steady power P_0 after t_{f}, plotted for the ring-up stage with different protocol times t_{f}. For comparison, we also plot a lower bound on P_{max}, which follows from Eq. 44 and the fact that $mP_{\text{max}} \geq \sum_{i=1}^{m} |\epsilon_i|^2 \geq E_{\text{min}}$:

$$P_{\text{max}}(\{x_i\}) \geq \frac{1}{m} \sum_{i=1}^{n} \left| \frac{(\mathbf{U^{-1}y})_\mathbf{i}}{s_i} \right|^2 \equiv P_{\text{max,lb}} \tag{45}$$

Given the protocol time $t_{\text{f}} = 60\,\text{ns} \approx 1/\kappa_{\text{r}}^0$ used in the main text, we find $P_{\text{max}} = 14.5\,\text{dB}$ after numerical optimization, which is a 4.1 dB reduction from that of the minimum energy pulse. For speedup beyond unit resonator lifetime $1/\kappa_{\text{r}}^0$, P_{max} grows rapidly and may induce

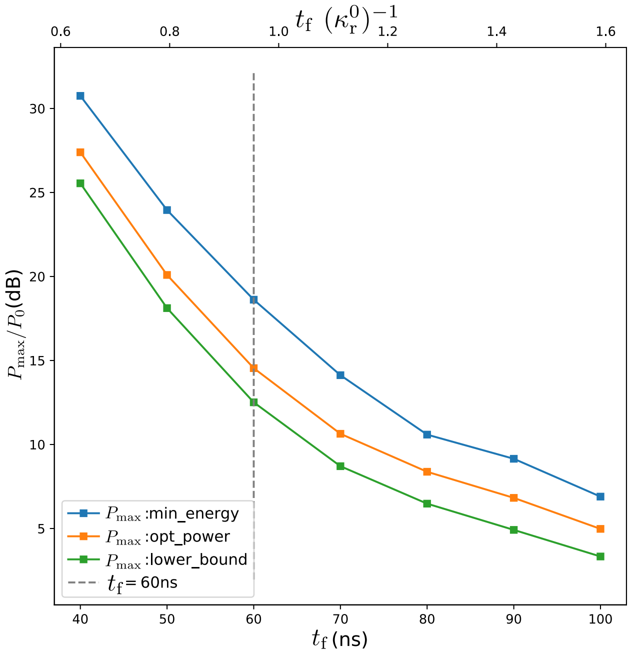

Supplementary Figure 3. **Speed-power relation for the MMOC protocol.** Maximum output power $P_{\max}$ (in dB) consumed, in units of the steady output power P_0 after t_f, plotted for different pulses and final times t_f. (Blue) The minimum energy pulse, by setting $x_i = 0$ in Eq. 42. (Orange) The pulse whose maximum power is numerically minimized, by exploiting the redundant degrees of freedoms x_i. (Green) Theoretical lower bound obtained from Eq. 45. The experimental parameters are the same as in the main text.

unwanted qubit transitions, which sets a speed limit for the MMOC protocol, as discussed in SN 7.

Computational complexity. For single-port MMOC, the number of total qubit-state-conditioned bosonic modes n (in our experiment $n = 4$) is less than the total number m of pulse sections. In this case, the most time-consuming step in computing Eq. 42 is the singular value decomposition of the $m \times n$ matrix $\mathbf{G}$, which has time complexity $O(mn \min(m, n)) = O(mn^2)$. For the general case of n-port driving, G is replaced by the $n \times mn$ matrix M, with corresponding complexity $O(mn^3)$. In either case, the problem admits an efficient polynomial time solution.

SUPPLEMENTARY NOTE 7. INFLUENCES OF THE LARGE DRIVE

We observe that the output signal drifts with a large driving power, which sets a limit on the steady-state driving power of our protocols. This can be explained by the nonlinearity of the resonator [12]. At the same time, according to the previous study [13], higher transmon levels are excited due to the non-RWA part of the qubit-resonator Hamiltonian, which becomes on-resonant as the photon number in the resonator increases through a Raman-like process. These two observations are shown to be closely related in theoretical simulations [14]. Here, we conduct two different experiments to confirm this point and find limitations of our protocol when applied to the transmon-resonator cQED system.

In the first experiment (Fig. 4), we compare the output signal of a small pulse of strength 1 a.u., and another larger pulse of strength 2.66 a.u.. Each point is averaged over 3×10^4 measurements and moving averaged with a Savitzky-Golay filter (width 21, order 3). IQ traces of the output signal in Fig. 4(c) show a clear drift even long after $5\kappa_\mathrm{a}^{-1}$, which can be qualitatively explained by the nonlinearity of the cavity mode. In Fig. 5 and 6, another experiment is conducted to test the impact on the transition out of the $|0\rangle$ state of different pulse amplitudes and durations. A significant drop in P_0 is observed above amplitude 1 a.u.. At this drive amplitude we estimate the steady-state cavity photon number (via qubit spectroscopy) to be roughly the critical photon number $n_\mathrm{c} \equiv (\Delta/2g)^2 \approx 18$ [15].

SUPPLEMENTARY NOTE 8. PROPAGATOR CORRECTIONS FROM THE LOW-PASS FILTER IN THE AWG DRIVING LINE

Here we show that corrections to the MMOC pulses imposed by the low-pass fourth-order Chebyshev filter are negligible. In our experiments, the MMOC pulse (Eq. 42) from the arbitrary wave generator (AWG) has a carrier driving frequency $\omega_\mathrm{s}/(2\pi)$ of 200 to 250 MHz, which passes through the filter with a cutoff frequency $\omega_\mathrm{c}/(2\pi) = 750\,\mathrm{MHz}$. The piece-wise constant pulse causes the Gibbs phenomenon, a potential source of error.

Here we give a qualitative evaluation of this error. To simplify our calculations, we assume that the passband's transfer function g is 1, and is 0 outside the passband. The waveform after the filter $\epsilon'(t)$ is described by a convolution $\mathcal{F}$ of the pre-filter waveform $\epsilon(t)$ with the

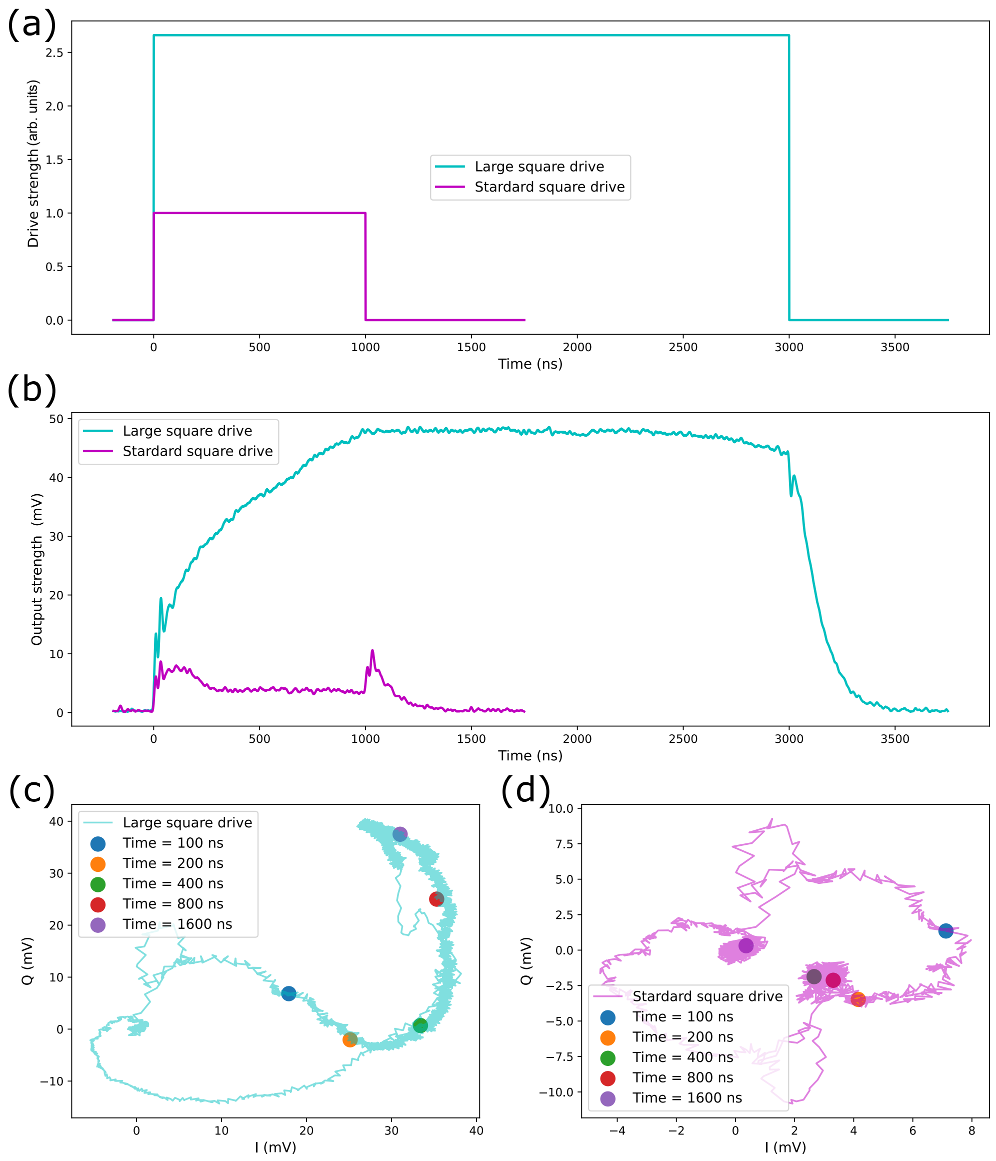

Supplementary Figure 4. **Output signal comparison for different pulse amplitudes.** (a) The square-wave drive pulse with large (blue) and small (pink) amplitudes. (b) The average output signal (in mV) for the two pulses, as a function of time. For the small amplitude pulse, steady output is reached after the expected equilibrium time $t_e = 5\kappa_r^{-1} \approx 300\,\text{ns}$. For the large amplitude pulse, the output signal continues to grow well beyond this time. (c,d) The I/Q quadrature trajectories for the (c) large and (d) small amplitude pulse. Various intermediate times are colour-coded in the figure.

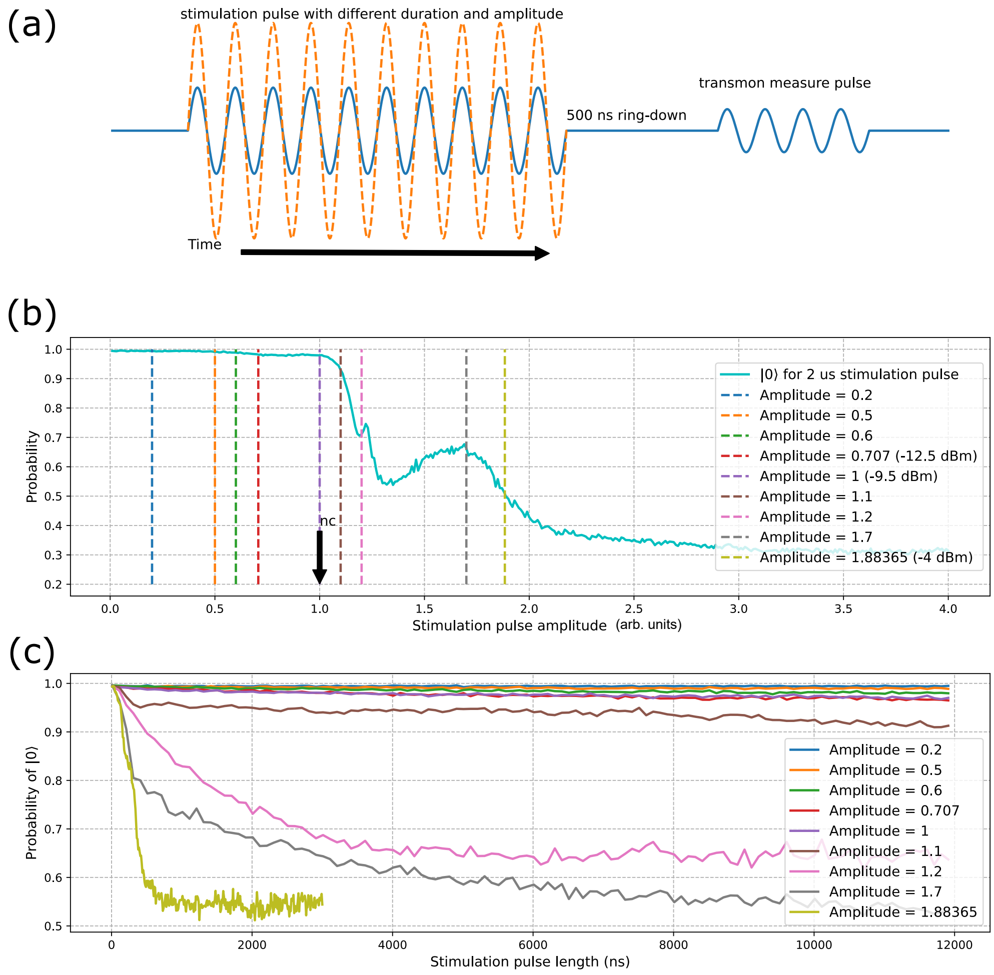

Supplementary Figure 5. **Impacts on qubit population induced by resonator excitation.** (a) Pulse sequence for the readout fidelity measurement. A stimulation pulse is first applied with variable strength and duration. After a 500 ns resonator ring-down, a weak measurement pulse is applied to measure the qubit state. (b) Readout fidelity for qubit ground state, P_0, as a function of stimulation pulse amplitude. The pulse duration is fixed at 2 μs. A resonance peak (of error) is found between amplitude 1 a.u. to 1.5 a.u., similar to the observations of [13]. (c) P_0 versus stimulation pulse duration, plotted for various amplitudes marked by coloured lines in (b). The fidelity drops drastically in the first 500 ns when the amplitudes are greater than 1 a.u.

filter function

$$\epsilon'(t) = \mathcal{F}[\epsilon](t) = \int_{-\infty}^{\infty} \epsilon(\tau) g(t-\tau) d\tau = \int_{-\infty}^{\infty} \epsilon(\tau) d\tau \int_{\omega_0}^{\omega_1} \frac{d\omega}{2\pi} e^{-i\omega(t-\tau)}, \tag{46}$$

with frequency cutoffs $\omega_0 = \omega_\mathrm{s} - \omega_\mathrm{c}$ and $\omega_1 = \omega_\mathrm{s} + \omega_\mathrm{c}$. The calculation is done in the rotating

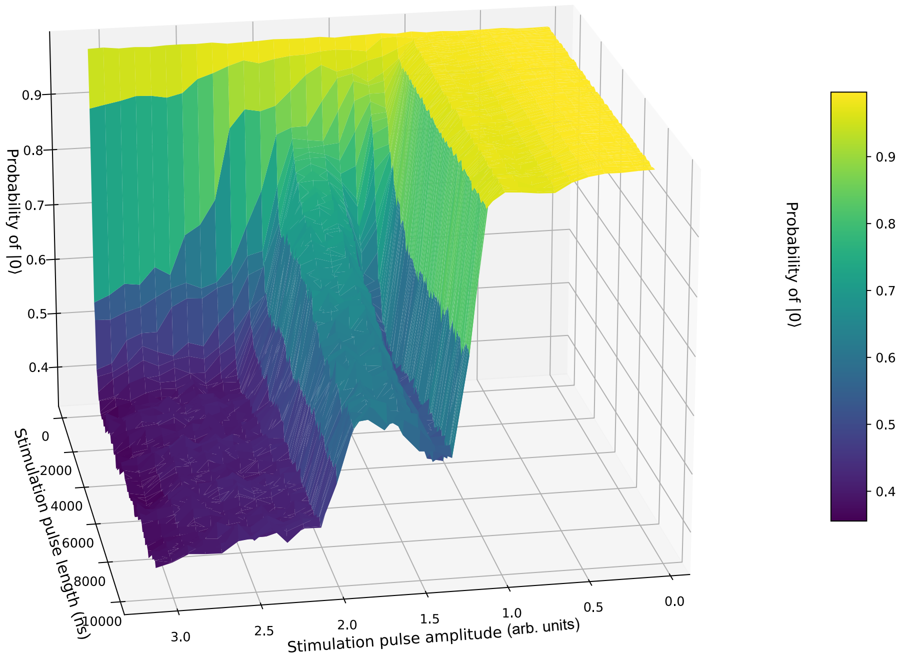

Supplementary Figure 6. **Impacts on qubit population induced by cavity excitation, the full data.** Population P_0 of the $|0\rangle$ state, measured as a function of stimulation amplitude and duration, following the procedures in Fig. 5. A clear drop in P_0 is found for amplitude greater than 1 a.u..

frame defined by ω_s. Replacing ϵ by ϵ' in Eq. 41 results in a modified constraint matrix $\mathbf{G}'$, given by $\mathbf{G}'\epsilon = \mathbf{G}\epsilon'$, which satisfies

$$G'_{ij} = \int_{t_0}^{t_1} \mathcal{F}[\theta_j](t) e^{-i\tilde{\Delta}'_i(t_\mathrm{f}-t)} dt \tag{47}$$

$$= \int_{t_{j-1}}^{t_j} e^{-i\tilde{\Delta}'_i(t_\mathrm{f}-\tau)} d\tau \int_{\omega_0}^{\omega_1} \frac{d\omega}{2\pi} \frac{e^{i(\tilde{\Delta}'_i-\omega)(t_1-\tau)} - e^{i(\tilde{\Delta}'_i-\omega)(t_0-\tau)}}{i(\tilde{\Delta}'_i-\omega)} \tag{48}$$

$$= e^{-i\tilde{\Delta}'_i t_\mathrm{f}} \int_{\omega_0}^{\omega_1} \frac{d\omega}{2\pi} \frac{e^{i\omega t_j} - e^{i\omega t_{j-1}}}{i\omega} \frac{e^{i(\tilde{\Delta}'_i-\omega)t_1} - e^{i(\tilde{\Delta}'_i-\omega)t_0}}{i(\tilde{\Delta}'_i-\omega)}, \tag{49}$$

where $[t_0, t_1]$ is the finite time window in which the corrected pulse ϵ' is integrated over, and $\theta_j(t)$ is the jth square function which equals 1 for $t_{j-1} \leq t \leq t_j$ and 0 elsewhere. The single integral Eq. 49 is easy to evaluate numerically and should be compared to the original

matrix elements G_{ij}. We find that the relative difference between G'_{ij} and G_{ij} is less than 10^{-6} and is generally independent of the choice of window time t_0, t_1.

SUPPLEMENTARY NOTE 9. ADDITIONAL EXPERIMENTAL DATA

In this section, we show additional experimental data we have collected. In Fig. 7, we test the widely used square wave driving with an initial amplitude twice as large as the remaining waveform, and see little decrease in the equilibrium time. In Fig. 8, we apply the CD pulse designed according to the set of parameters for the $|0\rangle$ and $|1\rangle$ states, and confirm that in the single-port driving situation, CD is only able to accelerate one mode at one time. The main text shows the 4-mode MMOC protocol controlling all four modes with the same pulse. In Fig. 9, we design the MMOC for two modes corresponding to only one specific qubit state, then apply it on both qubit states. In this case, the method only works when the qubit is in the correct state. In Fig. 10, we compare the output amplitudes of the $\sin^2$ and the corresponding CD ringup drives of different durations. Fig. 11 and Fig. 12 show the output signal's IQ trajectories for the $\sin^2$ and the corresponding CD ringup drives. In Fig. 13, a large-amplitude and far off-resonant $\sin^2$ drive , and corresponding CD drive are applied. The far-detuning guarantees the cQED system will not be excited. According to the input-output theory, the output signal will be a simple rescaling of the input signal. The designed input ringup duration t_f is 30 ns. However, we see the output reaches the designed stable value around $t = 65$ ns. This tail indicates the filtering effect of some low-Q and energy-storing microwave components in the feedline. This effect is more noticeable when the input signal is larger and can be simulated by a convolution with a low-pass filter transfer function.

To understand the effect of MMOC on qubit readout, we also apply MMOC to the rising edge of the readout pulse and compare the resulting readout fidelity with that corresponding to the regular rectangular pulse. Results are shown in Fig. 14 and Fig. 15. We find that MMOC improves the readout fidelity of $|0\rangle$ but decreases the readout fidelity of $|1\rangle$ due to T1 decay that occurs during the MMOC pulse. Longer T1 and a Bayesian-based algorithm will mitigate this error by correcting the bit flipping during the readout pulse [16] (experiment in progress).

Finally, by setting the decay rate $\kappa_\mathrm{r}^0 = 0$, we compare open system CD with closed system

CD. Results are shown in Fig. 16. As expected, we find that only the open system CD pulse accelerates the transition to equilibrium. That is because κ_{r}^{0} and the drive detuning are of a similar magnitude.

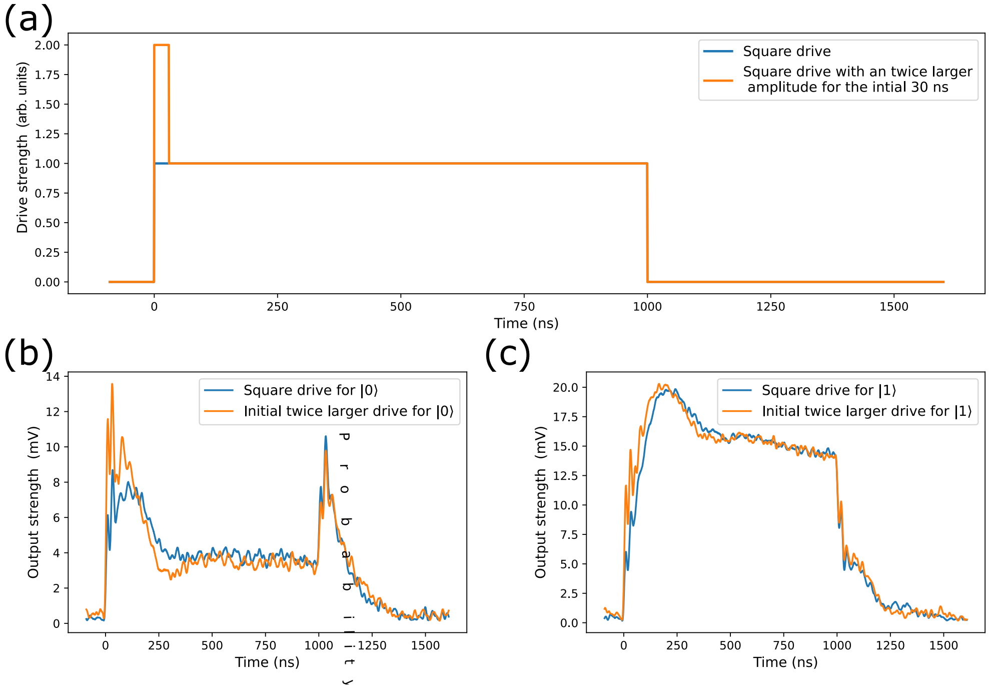

Supplementary Figure 7. **Initial larger pulse.** (a) Waveform comparison between the normal driving pulse and a driving pulse with an amplitude twice larger during the initial 30 ns. In (b) and (c), we see that the initial larger driving pulse does not make the equilibrium process much faster.

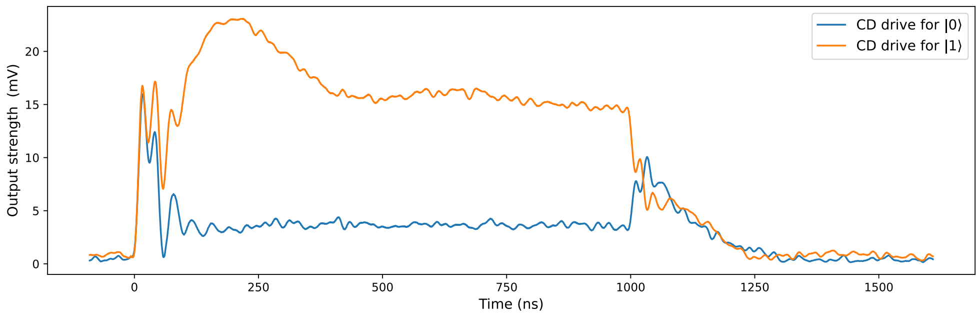

Supplementary Figure 8. Apply the CD driving designed for $|0\rangle$ on $|1\rangle$ state. The driving pulse for $|0\rangle$ is the same as the one used in Fig. 2 in the main text. The same pulse applied when the qubit is prepared in the $|1\rangle$ state will not accelerate the evolution to equilibrium.

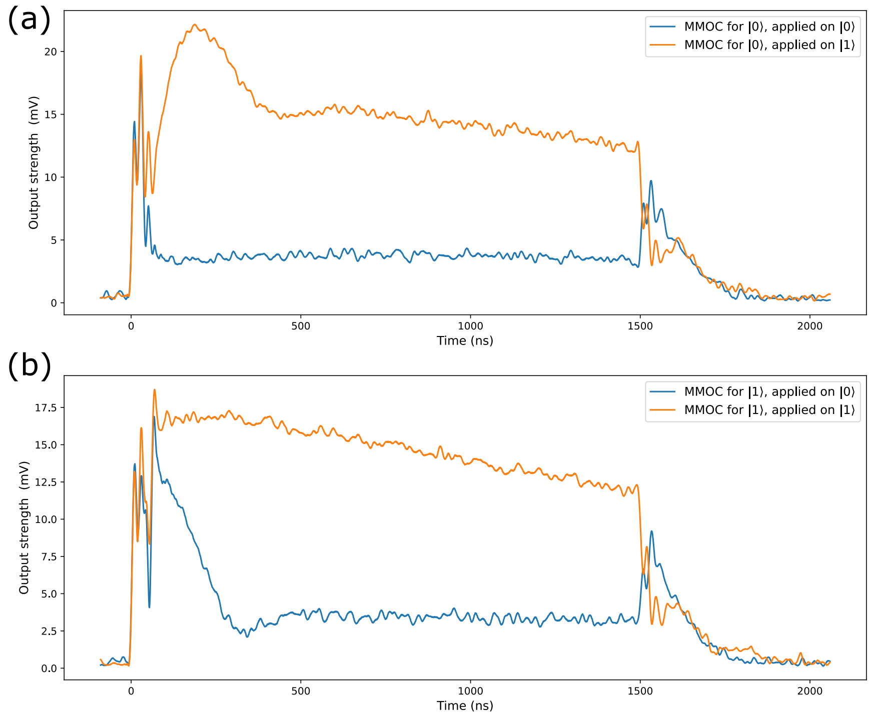

Supplementary Figure 9. Applying the multi-section MMOC designed for $|0\rangle(|1\rangle)$ when the qubit is in the $|1\rangle(|0\rangle)$ state. In addition to the 4-mode MMOC we used in the main text, we also test the 2-mode MMOC, designed for the hybrid resonator and filter modes with a fixed qubit state. In (a) and (b), we show the performance of the MMOC for a specific qubit state on both states and see that, as expected, the performance is worse when applied with the qubit in the other state. In (b), the decay of the output from ≈ 100 for $|1\rangle$ is caused by the limited lifetime of the qubit.

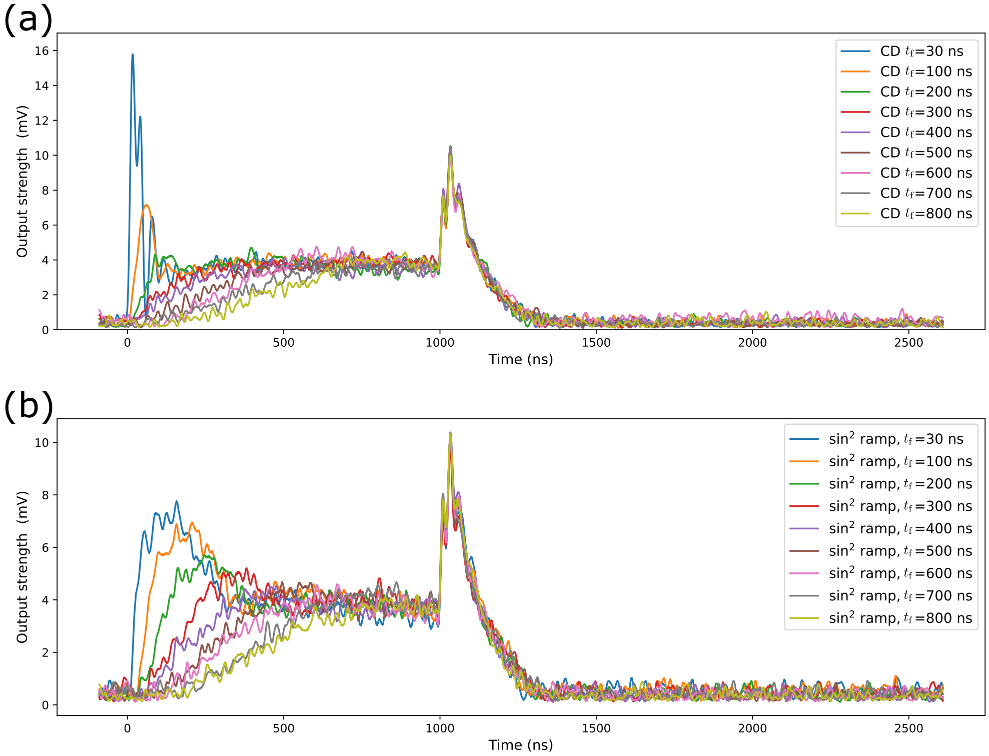

Supplementary Figure 10. **CD and** $\sin^2$ **ringup drives for different durations.** Output signal strength for CD and $\sin^2$ pulses with different ring-up durations, using the same parameters and experimental setup as in Fig. 2 of the main text.

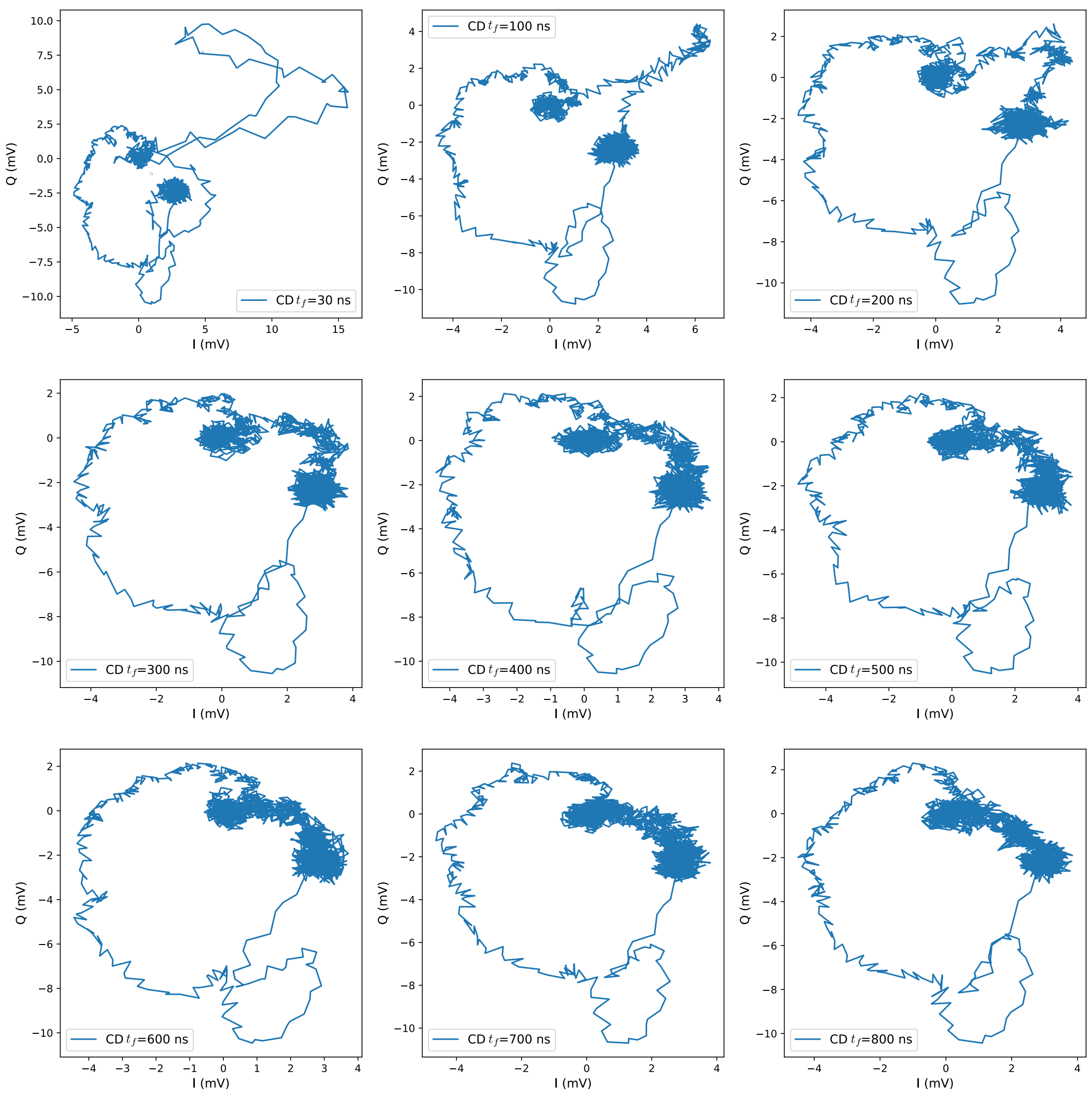

Supplementary Figure 11. **I-Q plots of CD driving for different ring-up durations.** We see a huge spike during the ringup for a short CD driving, which does not mean the system undergoes highly non-equilibrium dynamics. According to the input-output theory, the monitored IQ is the coherent superposition of the input driving and the leakage of the system. When the ringup is only 30 ns, the large amplitude will excite the untargeted filter mode, which is indicated by the spiral collapse to the equilibrium after 30 ns.

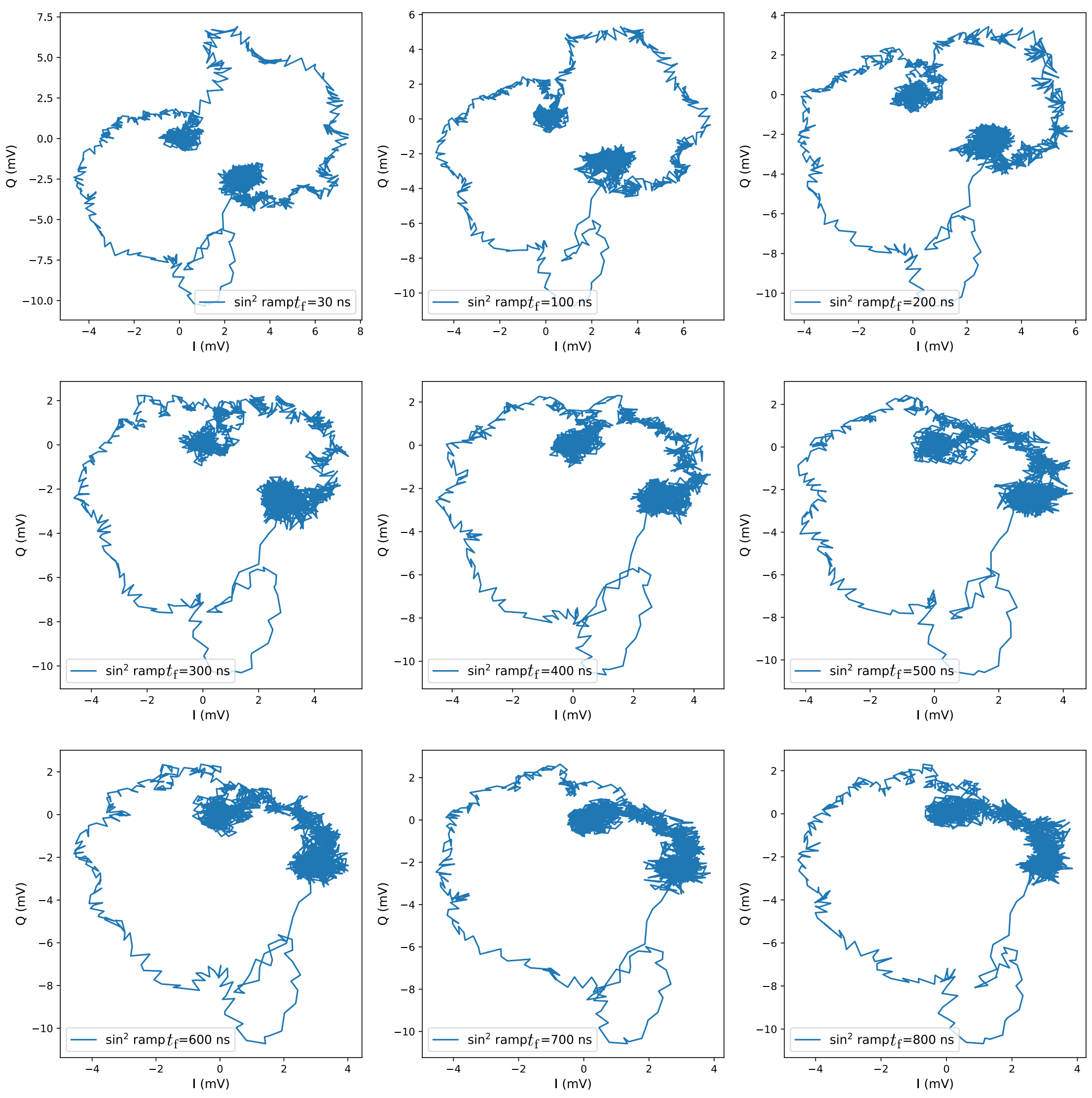

Supplementary Figure 12. **I-Q plots of the** $\sin^2$ **drive for different ringup durations.** To see how long it takes to reach quasi-equilibrium dynamics for the corresponding bare $\sin^2$ ringup, we measure the IQ trajectories for different t_{f}. Until $t_{\mathrm{f}} = 800\,\mathrm{ns}$, the trajectory will not change in shape, which indicates the system has reached the quasi-equilibrium dynamics. Compared this with the $< 100\,\mathrm{ns}$ in the case of CD driving.

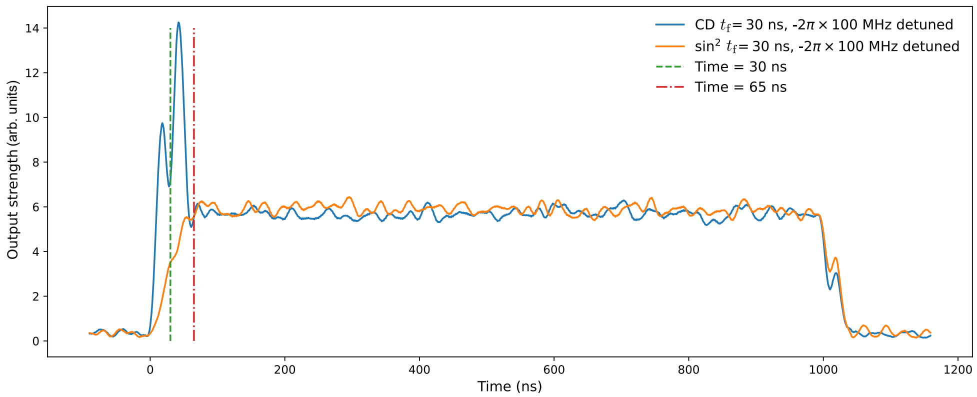

Supplementary Figure 13. **Output response of the** $-2\pi \times 100\,\mathbf{MHz}$ **detuned CD and** $\sin^2$ **large-amplitude drivings.** When we detune the drive frequency by $-2\pi \times 100\,\mathrm{MHz}$, the lossy cQED system will not be excited, and the output signal closely follows the input signal. However, we see a delay in the output signal before reaching equilibrium after ringup, and an extended ringdown after the drive has concluded. This is particularly pronounced when the driving power is large.

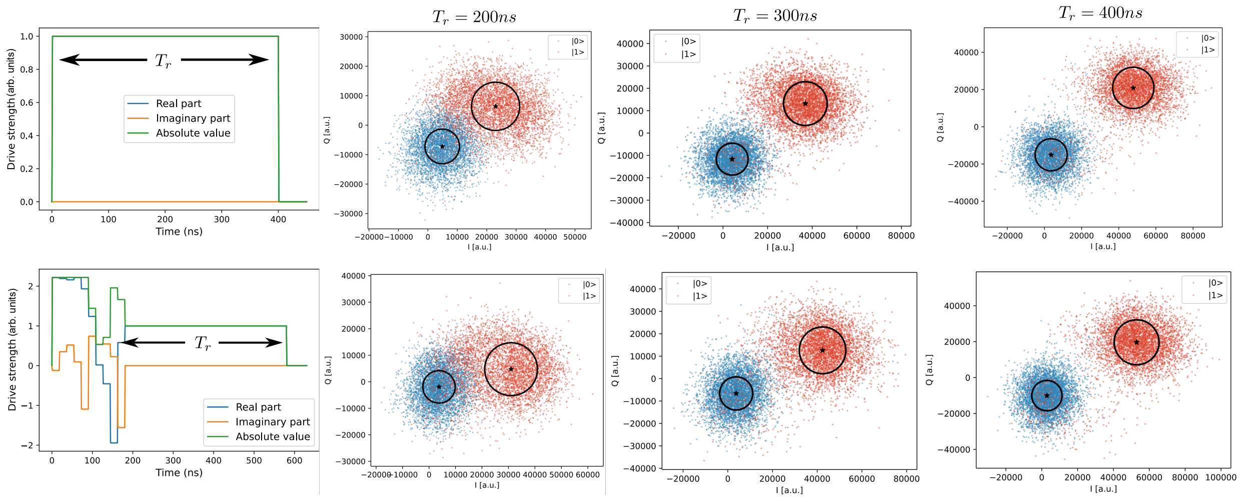

Supplementary Figure 14. **IQ for rectangular and MMOC ringups with different readout durations T_r.** To understand how MMOC affects qubit readout, we apply MMOC in the ringup process and compare the resulting IQ plot with that the one corresponding to a regular rectangular pulse. To prevent the large amplitude MMOC pulses from causing unwanted qubit excitations, we extend the duration of MMOC to 180 ns. IQ points are sampled only during the readout duration T_r to avoid capturing wild IQ trajectories during MMOC. The top row corresponds to rectangular pulses, and the lower row corresponds to MMOC ringup. The black point and circle in the IQ plots for each qubit state indicate the fitted center and one standard deviation over 5,000 repetitions.

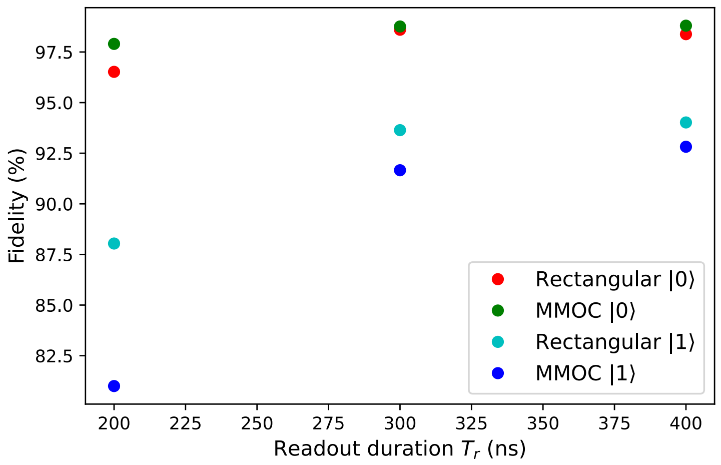

Supplementary Figure 15. **Readout fidelity for rectangular and MMOC ringup for different readout durations T_r.** Based on the IQ results of Supplementary Figure 14, we calculate the readout fidelity of different ringups for the qubit $|0\rangle$ and $|1\rangle$ states. MMOC leads to improved readout fidelity of $|0\rangle$ but decreased readout fidelity of $|1\rangle$. This can be explained as follows. In the case of $|0\rangle$, after the MMOC ringup, the IQ plot is more concentrated because the resonator is already in a steady state. If we apply the rectangular pulse, the natural charging duration $(5/\kappa)$ is about 350 ns, which means the IQ trajectory keeps drifting during the readout. In the case of $|1\rangle$, the main limitation is the T1 decay that occurs during the 180 ns MMOC pulse. One possible solution to this is to reduce the duration of the MMOC pulse, taking care to ensure that the pulse is not so short so as to lead to a maximum amplitude large enough that causes unwanted qubit excitations.

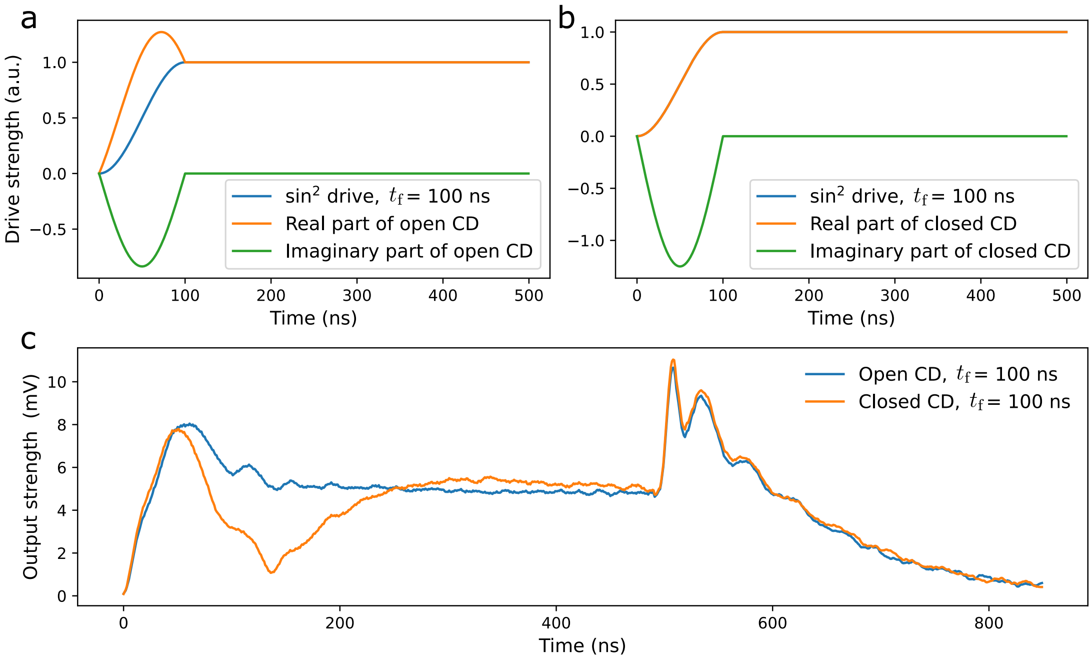

Supplementary Figure 16. **Comparison of open and closed system CD.** The original drive has a $\sin^2$-shape rising edge with a duration of 100 ns and lasts for 400 ns. (a) We calculate the open system CD waveform according to Eq. 1 in the main text. The resonator frequency $\omega_\mathrm{r}^0/(2\pi) = 6.4415$ GHz, drive frequency $\omega_\mathrm{s}/(2\pi) = 6.4395$ GHz, and decay rate $\kappa_\mathrm{r}^0 = 1/(56.6\,\mathrm{ns})$. (b) The closed system CD waveform is obtained by setting $\kappa_\mathrm{r}^0 = 0$. The real part of the waveform overlaps with the original $\sin^2$ drive. (c) Comparison of the the output amplitudes resulting from the two CD drives. Off-resonant coupling to the filter mode at 100 ns causes a small ripple in the open CD response, and we can reduce this with a smaller driving amplitude. The closed system CD drive does not accelerate the transition to equilibrium (see Fig. 2 in the main text which shows that it takes roughly 300 ns for a bare 100 ns $\sin^2$ pulse to cause the resonator to reach equilibrium). Measurements are repeated 30,000 times.

SUPPLEMENTARY REFERENCES

* shuomingan@tencent.com

[1] J. Heinsoo, C. K. Andersen, A. Remm, S. Krinner, T. Walter, Y. Salathé, S. Gasparinetti, J.-C. Besse, A. Potočnik, A. Wallraff, *et al.*, Rapid high-fidelity multiplexed readout of superconducting qubits, Physical Review Applied **10**, 034040 (2018).

[2] G. Vacanti, R. Fazio, S. Montangero, G. Palma, M. Paternostro, and V. Vedral, Transitionless quantum driving in open quantum systems, New Journal of Physics **16**, 053017 (2014).

[3] S. Wu, X. Huang, H. Li, X. Yi, *et al.*, Adiabatic evolution of decoherence-free subspaces and its shortcuts, Physical Review A **96**, 042104 (2017).

[4] M. Sarandy and D. Lidar, Adiabatic approximation in open quantum systems, Physical Review A **71**, 012331 (2005).

[5] D. F. Walls and G. J. Milburn, *Quantum optics* (Springer Science & Business Media, 2007).

[6] H.-P. Breuer, F. Petruccione, *et al.*, *The theory of open quantum systems* (Oxford University Press on Demand, 2002).

[7] D. Bures, An extension of kakutani's theorem on infinite product measures to the tensor product of semifinite w*-algebras, Transactions of the American Mathematical Society **135**, 199 (1969).

[8] S. L. Braunstein and C. M. Caves, Statistical distance and the geometry of quantum states, Physical Review Letters **72**, 3439 (1994).

[9] L. Mandelstam and I. Tamm, The uncertainty relation between energy and time in non-relativistic quantum mechanics, in *Selected papers* (Springer, 1991) pp. 115–123.

[10] M. M. Taddei, B. M. Escher, L. Davidovich, and R. L. de Matos Filho, Quantum speed limit for physical processes, Physical review letters **110**, 050402 (2013).

[11] C. W. Gardiner and M. J. Collett, Input and output in damped quantum systems: Quantum stochastic differential equations and the master equation, Physical Review A **31**, 3761 (1985).

[12] P. Brookes, G. Tancredi, A. D. Patterson, J. Rahamim, M. Esposito, T. K. Mavrogordatos, P. J. Leek, E. Ginossar, and M. H. Szymanska, Critical slowing down in circuit quantum electrodynamics, Science advances **7**, eabe9492 (2021).

[13] D. Sank, Z. Chen, M. Khezri, J. Kelly, R. Barends, B. Campbell, Y. Chen, B. Chiaro, A. Dunsworth, A. Fowler, *et al.*, Measurement-induced state transitions in a superconducting qubit: Beyond the rotating wave approximation, Physical review letters **117**, 190503 (2016).

[14] T. K. Mavrogordatos, G. Tancredi, M. Elliott, M. Peterer, A. Patterson, J. Rahamim, P. Leek, E. Ginossar, and M. Szymańska, Simultaneous bistability of a qubit and resonator in circuit quantum electrodynamics, Physical review letters **118**, 040402 (2017).

[15] A. Blais, R.-S. Huang, A. Wallraff, S. M. Girvin, and R. J. Schoelkopf, Cavity quantum electrodynamics for superconducting electrical circuits: An architecture for quantum computation, Physical Review A **69**, 062320 (2004).

[16] J. Gambetta, W. Braff, A. Wallraff, S. Girvin, and R. Schoelkopf, Protocols for optimal readout of qubits using a continuous quantum nondemolition measurement, Physical Review A **76**, 012325 (2007).
